# Supplementary material for: A molecular epidemiological analysis of Burkholderia pseudomallei in southern Thailand
Source: PLoS Negl Trop Dis. 2024 Aug 22;18(8):e0012444. doi: 10.1371/journal.pntd.0012444 (PMC11373835; doi:10.1371/journal.pntd.0012444)
Supplement: Attachment — Submitted filename: Authors responses to reviewers comments.docx [file pntd.0012444.s003.docx]

**Authors’ responses to the reviewers’ comments and concerns**

**Original letter from the editors:**

Dear Dr. Tuanyok,
 
Thank you very much for submitting your manuscript "A molecular epidemiological analysis of Burkholderia pseudomallei in southern Thailand." for consideration at PLOS Neglected Tropical Diseases. As with all papers reviewed by the journal, your manuscript was reviewed by members of the editorial board and by several independent reviewers. In light of the reviews (below this email), we would like to invite the resubmission of a significantly-revised version that takes into account the reviewers' comments.
 
The reviewers felt this was a well-performed study with strong results and of public health importance. However, it was noted that complete NGS data is available for these samples, but they have been analysed only by MLST. Given that the NGS data is already available, the samples can be also analysed at the SNP level, which would allow you to answer some of the questions posed by reviewer #1 and would strengthen the conclusions of your paper. For this reason I have recommended a major revision to incorporate whole genome SNP analysis into this study.

We cannot make any decision about publication until we have seen the revised manuscript and your response to the reviewers' comments. Your revised manuscript is also likely to be sent to reviewers for further evaluation.
 
When you are ready to resubmit, please upload the following:

[1] A letter containing a detailed list of your responses to the review comments and a description of the changes you have made in the manuscript. Please note while forming your response, if your article is accepted, you may have the opportunity to make the peer review history publicly available. The record will include editor decision letters (with reviews) and your responses to reviewer comments. If eligible, we will contact you to opt in or out.

[2] Two versions of the revised manuscript: one with either highlights or tracked changes denoting where the text has been changed; the other a clean version (uploaded as the manuscript file).

Important additional instructions are given below your reviewer comments.

Please prepare and submit your revised manuscript within 60 days. If you anticipate any delay, please let us know the expected resubmission date by replying to this email. Please note that revised manuscripts received after the 60-day due date may require evaluation and peer review similar to newly submitted manuscripts.
 
Thank you again for your submission. We hope that our editorial process has been constructive so far, and we welcome your feedback at any time. Please don't hesitate to contact us if you have any questions or comments.
 
Sincerely,

Elizabeth M Batty
Academic Editor
PLOS Neglected Tropical Diseases

Stuart Blacksell
Section Editor
PLOS Neglected Tropical Diseases

***********************
A brief response to the editors’ letter

Dear Editors,

We greatly appreciate the suggestions from the editors and the comments from the reviewers. This study primarily focused on the regional diversity of *Burkholderia pseudomallei* in southern Thailand, utilizing available genetic databases such as MLST from known strains in the historic Finkelstein collection and recent human melioidosis cases. Therefore, we chose to present only MLST data in this study.

In our forthcoming follow-up studies, we are sequencing more *B. pseudomallei* strains isolated from human and animal cases, as well as from the environment. At that time, we will perform whole genome SNP analysis and global phylogenetic analyses. We understand the research community's interest in further insights into *B. pseudomallei* strains from southern Thailand. In the meantime, we have released all 126 genomes of B. pseudomallei strains sequenced in this study through GenBank as the Sequence Read Archive (SRA) under the BioProject accession no. PRJNA1113702.

Thank you for your consideration.

Sincerely,

Apichai Tuanyok, Ph.D., and Kwanjit Duangsonk, Ph.D.

Co-corresponding authors

**Reviewer's Responses to Questions**

Key Review Criteria Required for Acceptance?
As you describe the new analyses required for acceptance, please consider the following:

**Methods**
-Are the objectives of the study clearly articulated with a clear testable hypothesis stated?
-Is the study design appropriate to address the stated objectives?
-Is the population clearly described and appropriate for the hypothesis being tested?
-Is the sample size sufficient to ensure adequate power to address the hypothesis being tested?
-Was correct statistical analysis used to support conclusions?
-Are there concerns about ethical or regulatory requirements being met?

**Reviewer #1**: Generally: The design of the study is comprehensible and the methods chosen are largely appropriate. However, my main concern is the use of MLST analysis, SNP analyses should be performed for the comparison of related isolates. The necessary data are available, which is why it is surprising that they are only used for the assignment of the ST.

**Authors:** We appreciate the reviewer's comment and concern regarding our choice of MLST analysis over SNP analysis. Our decision to use MLST was influenced by the historical context of the Finkelstein collection, where the *B. pseudomallei* strains had not been sequenced, and only their sequence types (STs) analyzed by traditional MLST were available. Therefore, we utilized MLST data from our recent human isolates and the STs extracted from available whole-genome sequencing (WGS) data to compare with those from the Finkelstein collection.

In our ongoing research, we are sequencing additional strains from southern Thailand, including isolates from animal cases and environmental sources. We have also recently obtained the original stocks of Finkelstein's strains from the CDC, which we plan to sequence as well. Although SNP analysis was not feasible for the current study, it is a planned component of our future research to provide more detailed genetic comparisons.

We apologize for any disappointment this may cause and appreciate the suggestion. We are committed to incorporating SNP analysis in our future studies to enhance the robustness of our findings.

•       There is a discrepancy in the numbers see line 38 and line 119.

**Authors:** Thank you for pointing out the discrepancy. We have corrected this discrepancy. It now reads, “In this study, 263 *B. pseudomallei* isolates from 156 patients were used”.

•       Line 117: “Multiple B. pseudomallei isolates from different diagnosed specimens were collected from each patient” and Line 240: "To study the strain diversity in individual infection, at least two B. pseudomallei isolates were collected from cultures of available clinical specimens (e.g. blood, sputum, pus, urine) in 59 patients. **"**
The authors write that they are investigating the genetic diversity of pseudomallei in patient samples. However, it is not clear to me how the isolates were selected. Were all isolates analyzed here, or a few random ones? Please clarify the sentences above. What was the basis on which the strains were selected if not all were analyzed.

Authors: We appreciate the reviewer’s question regarding the selection of *B. pseudomallei* isolates for analysis. In this study, we analyzed at least one isolate from each of the 158 patients. We have also mentioned in the manuscript that “majority of these isolates were from cases identified during 2014 and 2017”. This means that the isolates from that time period were used in the analysis. However, recent isolates from cases in 2018 and 2020 were randomly included. The revised statements now read ”Of note, majority of these isolates were from cases identified during 2014 and 2017. Recent isolates from cases in 2018 and 2020 were also randomly selected”,

For 59 patients, where isolates were available from cultures of multiple clinical specimens (e.g., blood, sputum, pus, urine), we included more than one isolate in the analysis to study the genetic diversity within each patient. The selection was based on the availability of isolates from different specimen types to provide a comprehensive understanding of the strain diversity in individual infections.

•       The methods for the detection of B. pseudomallei in the environment sound plausible. Do they correspond to those used in the field or to the consensus guidelines? The authors might provide citations for clarification.

**Authors:** We appreciate the reviewer's inquiry regarding our methods for detecting *B. pseudomallei* in the environment. We followed the consensus guidelines with a minor modification, such as using gentamicin instead of colistin in the enrichment broth. This modification was made to maintain consistency in antibiotic selection between the enrichment broth and Ashdown’s agar. We chose gentamicin based on our observation that there were no reports of a gentamicin-susceptible phenotype of *B. pseudomallei* in Thailand.

Furthermore, the technique for isolating *B. pseudomallei* from water samples, which involved direct, filtrated, and enrichment cultures, was novel and not described in the consensus guidelines. We found this approach to yield good outcomes in our study.

To address the reviewer's suggestion, we have added a statement and provided a citation in the revised manuscript. It now reads, “Both the direct and the enrichment soil cultures described above followed the consensus guidelines [26], with the exception that gentamicin was used instead of colistin in the enrichment broth.”

•       Please, explain the purpose of the different filters for the water filtrate culture.

**Authors:** Thank you for inquiring about this aspect of our methodology. The use of different filters served distinct purposes in our water filtrate culture procedure. Firstly, we employed Whatman Grade 1 filter paper to capture sediments such as soil, sand, grass roots, and wood present in the water sample. Following this, the flow-through from the initial filtration was further processed using a 0.45 µm diameter - 47 mm filter membrane via a vacuum filtration system. This step aimed to isolate bacterial cells from the filtered liquid. Subsequently, both the sediment captured by the filter paper and the bacterial cells collected on the filter membrane were utilized in our water filtrate culture procedure.

•       Line 183: what enrichment broth was used?

**Authors:** Thank you for raising this question. The details of the enrichment broth used were provided previously under the section b) Enrichment culture, specifically at line 183 of the revised manuscript.

•       Line 186: “a) Direct filtrate culture” should be “B)” according to line 172 and Fig.1
as in Line 190: “b) Enriched filtrate culture” should be “C)” according to line 172 and Fig.1

**Authors:** Thank you for pointing out these inconsistencies. The corrections have been made accordingly.

•       Please deposit the acquired NGS data to public databases and provide the Accession No. in the manuscript

**Authors:** Thank you for the suggestion. The NGS data has been publicly released through GenBank as the Sequence Read Archive (SRA) under the BioProject accession number PRJNA1113702. You can access the data at the following link: <https://www.ncbi.nlm.nih.gov/sra/?term=PRJNA1113702>. The accession number has been added to the revised manuscript.

•       Line 214 – 218: In contrast to the other parts of the methods, too little information is contained here. Please provide names of the tools and parameters used for the generation of the assemblies.

**Authors:** Thank you for your comments. We have addressed this concern by adding detailed information about the genomic assemblies and an additional citation to the revised manuscript. The statements now read as follows:

“Genomic assemblies were performed using various programs on Galaxy, a web-based framework for biological computing at the University of Florida. Briefly, genome contigs were generated from the FASTQ files using a Galaxy workflow: FASTQ Groomer (Galaxy Version 1.1.1) and SPAdes genome assembler (Galaxy Version 3.12.0+galaxy1) with default kmers: 21, 35, and 55. Each genome contained 200-300 contigs with approximately 30-50X coverage. The local BLASTN function on BioEdit version 7.0.5.3 was used to search for MLST gene allele sequences [28]”

•       Major: The analysis should not be limited to MLST but should definitely be extended to an SNP analysis.

**Authors:** We appreciated the suggestion from the reviewer. However, the scope of this current is limited by the availability of DNA sequences for the strains in Finkelstein collection, which are not publicly available. Consequently, we were only able to utilize their MLST data for comparison purposes. As mentioned in our response to the reviewer's previous question, we are committed to conducting SNP analysis as part of a follow-up study with the goal of placing the genetics of the strains from southern Thailand within the global phylogeny of *B. pseudomallei*. We are currently in the process of sequencing additional strains from various sources under a One Health framework for the study of melioidosis in southern Thailand, and inviting experts in *Burkholderia* genetics to participate in this follow-up study. These efforts will include strains from nearby geographic regions such as Peninsular Malaysia and Singapore for comparative analysis. Importantly, our genome data have been publicly released, allowing melioidosis researchers to freely utilize these genomes to reconstruct phylogenetic trees and contribute to the broader understanding of *B. pseudomallei* diversity.

**Reviewer #2**: The experimental design appears sound, and the method section is well-articulated, covering most of the essential information. Nevertheless, a few issues require further attention.

Primarily, the authors mentioned "the whole genome sequencing data" in line 205 without providing a data availability statement. Additionally, in line 215, they referred to using various programs on Galaxy for assembly. However, they still need to elaborate on how they determined the optimal results using different programs or pipelines.

**Authors:** We appreciate the comments from Reviewer 2. These comments were also raised by Reviewer 1. In response, we have provided detailed information regarding the genome data availability and the assemblies using programs on Galaxy in the revised manuscript. The statements now read as follows:

“The sequencing data of 126 genomes of *B. pseudomallei* sequenced in this study are available as the GenBank’s Sequence Read Archive (SRA) under the BioProject accession number PRJNA1113702. Genomic assemblies were performed using programs on Galaxy, a web-based framework for biological computing at the University of Florida. Briefly, genome contigs were generated from the FASTQ files using a Galaxy workflow: FASTQ Groomer (Galaxy Version 1.1.1) and SPAdes genome assembler (Galaxy Version 3.12.0+galaxy1) with default kmers: 21, 35, and 55. Each genome contained 200-300 contigs with approximately 30-50X coverage. The local BLASTN function on BioEdit version 7.0.5.3 was used to search for MLST gene allele sequences [28]. The allele sequences of seven housekeeping genes including, *ace*, *gltB*, *gmhD*, *lepA*, *lipA*, *nark*, and *ndh* of *B. pseudomallei* K96243 (Sequence Type 10), were used as the query sequences. All seven housekeeping gene sequences extracted from these genome data were used in the analysis. New gene allele numbers and new sequence types were assigned by pubMLST (<https://pubmlst.org>).”

Secondly, the manuscript omits the in silico analysis of MLST, a crucial aspect of the method section. It would be beneficial to include these details in the manuscript.
**Authors:** Thank you for raising this concern. We have addressed this by adding details on how to extract the allele sequences of the seven housekeeping genes, as described above, in the revised manuscript.
--------------------

**Results**
-Does the analysis presented match the analysis plan?
-Are the results clearly and completely presented?
-Are the figures (Tables, Images) of sufficient quality for clarity?

**Reviewer #1**: In my opinion, analysis at ST level is not too informative here, especially since NGS data is available. Even with identical STs, major genetic differences would not be surprising. I recommend an analysis that makes use of the NGS data. A lot of interesting questions come to mind, e.g. How similar are patient isolates with the same ST - between patients and isolates from a single patient? How do patient isolates from the same patient with different STs compare? Are there any special genetic features of isolates with STs that occur more frequently in patients?

**Authors:** We appreciate the comments and suggestions from the reviewer. We agree that the NGS data would provide better resolution of the genetic diversity among the *B. pseudomallei* isolates being compared, and the findings could lead to new hypotheses, particularly if special genetic features are identified in isolates with STs that occur more frequently in patients. However, due to the scope limitations of this current study, we have included the reviewer’s comments for discussion in the revised manuscript. These points will warrant further investigation as a follow-up study by us or other researchers.

•       The reader should be introduced to BTFC&YLF genomic groups.

**Authors:** We appreciate the reviewer's suggestion. In response, we have provided a description of the BTFC&YLF genomic group in the instruction section of the revised manuscript. It now reads: "

“Additionally, we investigated specific genetic markers such as serogroups [22] and BTFC &YLF (*B. thailandensis*-like flagella and chemotaxis &*Yersinia*-like fimbrial gene clusters) genomic groups [23] to provide a more comprehensive understanding of the genetic diversity and distribution of *B. pseudomallei* in southern Thailand. These genomic groups, which have dissimilar geographic distributions, offer insights into the genetic and evolutionary differences among *B. pseudomallei* populations.”

•       Line 258: Please rephrase. 2 samples per location would be 416?

**Authors:** We apologize for the confusion. What we meant to convey is that, on average, two samples were tested positive from each positive sampling location. Therefore, the total of 116 positive samples from 52 positive sampling locations is accurate. To provide better clarification, we have modified these statements in the revised manuscript. It now reads: "Out of the total of 2737 soil samples collected from 208 sampling locations, 116 (4%) samples from 52 (25%) sampling locations yielded the presence of B. pseudomallei through culturing. On average, two soil samples from each of these positive sampling locations were tested positive for *B. pseudomallei*."

•       The paragraphs on the isolation of Bp from soil and water are somewhat redundant with the corresponding tables.

**Authors:** We appreciate the reviewer’s observation. While we acknowledge that some redundancy exists between the statements provided in the paragraphs and the information presented in Table 1, our intention was to highlight key findings for readers who may prefer to access the information in a narrative format rather than referring solely to the table. These statements offer a condensed overview of the results, which may be beneficial for some readers. Therefore, we have chosen to retain these statements in their current form.

•       The authors have characterized the soil samples in detail. However, the results remain very descriptive. Here the study might benefit from a more in-depth analysis.
**Authors:** We appreciate the reviewer's suggestion for a more in-depth analysis. While we acknowledge that the current results may appear descriptive, we are currently in the process of conducting a follow-up study to delve deeper into the analysis of these soil samples. Specifically, we aim to identify physical, chemical and biological factors, such as the presence of other microorganisms and bacteriophages, that may be associated with the presence of *B. pseudomallei*. We apologize if the current level of analysis may disappoint the reviewer, but we are grateful for the suggestion and will endeavor to address it in our future research.

**Reviewer #2:** The manuscript's data is meticulously organized and presented with utmost clarity, indicating a high proficiency level and an understanding of the subject matter. The authors have demonstrated exceptional attention to detail and have managed to present the information concisely and understandably. This level of organization and presentation is commendable and should be maintained in future work. Overall, the data presented in this manuscript is exemplary and highlights the author's capabilities in the field of study.
**Authors:** We sincerely appreciate Reviewer #2's positive feedback on the organization and clarity of our manuscript. It is gratifying to know that our efforts to present the data in a meticulous and understandable manner have been recognized. We are committed to maintaining this standard of organization and presentation in our future work. Thank you for acknowledging our capabilities in the field of study.
--------------------

**Conclusions**
-Are the conclusions supported by the data presented?
-Are the limitations of analysis clearly described?
-Do the authors discuss how these data can be helpful to advance our understanding of the topic under study?
-Is public health relevance addressed?

**Reviewer #1:** The relevance of the study for public health is evident.
Still, not only the results but also the conclusions that can be drawn will benefit from an SNP analysis.

**Authors:** We appreciate the reviewer’s comment and agree that SNP analysis would provide more detailed genetic insights. However, we believe that the current study, even though it utilizes MLST for genetic analysis, still offers valuable contributions to public health. The findings from MLST analysis are significant in understanding the genetic diversity and epidemiology of *B. pseudomallei*, and they lay a strong foundation for future studies that will incorporate SNP analysis to further enhance our understanding.

Line 407-408: An SNP analysis would substantiate the hypothesis (including strains from southern Thailand, Malaysia, Singapore and, for example, northeast Thailand in the analysis).

**Authors:** We appreciate the reviewer's suggestion to include an SNP analysis to substantiate our hypothesis. We agree that incorporating SNP analysis would provide a more robust genetic comparison and help to map *B. pseudomallei* strains from southern Thailand onto the global phylogeny more accurately. We have revised the manuscript to reflect the importance of SNP analysis for future research. The revised paragraph now reads:

"Given these common STs among *B. pseudomallei* strains in the Malay Peninsula, we hypothesize that most *B. pseudomallei* strains from southern Thailand are likely more closely related to those from Malaysia and Singapore than to those from Northeast Thailand, owing to geographic proximity. This suggests that they might fall within one of the Malay sub-region groups, which currently include only strains from Malaysia and Singapore in the global phylogeny, as previously described [17]. While our current study was based on MLST data, we believe that further investigation, including SNP-based analysis, is warranted to map *B. pseudomallei* strains from southern Thailand onto the global phylogeny. Future studies will include SNP analysis of strains from southern Thailand, Malaysia, Singapore, and Northeast Thailand to provide a more comprehensive understanding of their genetic relationships. "

Line 433: Although the proportions of B. pseudomallei-positive soil and water sources are similar (25% for soil versus 26% for water), we propose that melioidosis in Songkhla Province is predominantly waterborne.” Please explain why?

**Authors:** This is a good question. Although the proportions of *B. pseudomallei*-positive soil and water sources are similar, we propose that melioidosis in Songkhla Province is predominantly waterborne based on several key observations:

1. Low positive rate in rice fields: Only 9% of rice fields were positive for *B. pseudomallei*. This contrasts with the higher positive rates found in other water sources, suggesting that soil may not be the primary reservoir for the bacterium in this region.
2. Occupational exposure: Our previous report from the same province (Kaewrakmuk et al., 2023; ref. no. 9) indicated that only 21% of melioidosis patients were rice farmers or had occupations (e.g., construction workers, park rangers) involving daily soil/water contact. This further supports the notion that direct soil exposure may not be the main route of infection.
3. Association with rainfall and flooding: The same study demonstrated a strong association between melioidosis cases and rainfall or flooding events, which occur most years. Floodwaters in Songkhla Province come from streams, surface runoffs, and waterfalls, indicating that waterborne transmission is more likely during these periods.
4. Environmental sampling: The positive water sources, including streams and floodwaters, are more frequently in contact with the population, especially during flooding events, increasing the likelihood of waterborne transmission.

Based on these points, we believe that waterborne routes play a more significant role in the transmission of *B. pseudomallei* in Songkhla Province compared to soil exposure. These statements have been added to the revised manuscript. It now reads:

“Although the proportions of *B. pseudomallei*-positive soil and water sources are similar (25% for soil versus 26% for water), several observations lead us to propose that melioidosis in Songkhla Province is predominantly waterborne. Firstly, only 9% of rice fields tested positive for *B. pseudomallei*, indicating a lower prevalence of the bacterium in soil compared to water sources. Secondly, our previous study found that only 21% of melioidosis patients were rice farmers or had occupations involving daily soil/water contact, such as construction workers or park rangers [9]. This suggests that direct soil exposure is not the main route of infection for most patients. Thirdly, we observed a strong association between melioidosis cases and periods of rainfall or flooding, which occur frequently in the region. During these events, floodwaters originating from streams, surface runoffs, and waterfalls, are more likely to be contaminated with *B. pseudomallei*. These water sources are in frequent contact with the population during floods, increasing the likelihood of waterborne transmission. Collectively, these findings support our hypothesis that waterborne routes play a more significant role in the transmission of *B. pseudomallei* in Songkhla Province compared to soil exposure.”

**Reviewer #2:** The data that has been presented in this study provides strong evidence to support the conclusion that has been drawn. The findings have been conveyed through a well-structured discussion that includes a detailed analysis of the data, as well as a thorough examination of the relevant literature. The figures that have been included in the study are not only visually appealing but are also very easy to understand, making it easy for readers to grasp the findings of the study. Overall, this study has been conducted rigorously and systematically, and the results are highly credible and informative.
**Authors:** We sincerely appreciate the positive feedback from Reviewer #2. We are pleased to hear that our study's data, analysis, and presentation have been well-received. Your recognition of the structured discussion, detailed analysis, and the clarity of our figures is encouraging. We strive to maintain a high level of rigor and systematic approach in our research, and we are glad that our efforts have resulted in credible and informative findings. Thank you for your valuable comments and support.
--------------------

**Editorial and Data Presentation Modifications?**
Use  this section for editorial suggestions as well as relatively minor modifications of existing data that would enhance clarity. If the only modifications needed are minor and/or editorial, you may wish to recommend “Minor Revision” or “Accept”.

**Reviewer #1**: (No Response)

**Reviewer #2**: please review the comments for Methods
**Authors:** We appreciate the reviewer's comments regarding the Methods section. We have carefully addressed your concerns and suggestions in our responses above. We hope you find our explanations and revisions satisfactory. Thank you for your constructive feedback.
--------------------

**Summary and General Comments**
Use this section to provide overall comments, discuss strengths/weaknesses of the study, novelty, significance, general execution and scholarship. You may also include additional comments for the author, including concerns about dual publication, research ethics, or publication ethics. If requesting major revision, please articulate the new experiments that are needed.

**Reviewer #1:** In their study "A molecular epidemiological analysis of Burkholderia pseudomallei in southern Thailand." Jedsada Kaewrakmuk and colleagues report the results of their MLST-based typing of patient isolates of the pathogen B. pseudomallei from southern Thailand. The study is complemented by an extensive investigation of the prevalence of the pathogen in the environment. The study is certainly of relevance to policy makers and the melioidosis community, not only in Thailand but also in other endemic areas.
The introduction provides a good detailed overview of the pathogen, the disease and its prevalence and discusses the need and relevance of environmental screening in this region. The latter could be shortened somewhat. In my opinion, the introduction does not cover previous studies enough. E.g. what is the current state of the literature on genetic studies on B. pseudomallei (MLST, or also SNP-based)? The same applies to the environmental prevalence of B. pseudomallei and studies comparing patients and environmental isolates. Finally, the introduction should also address factors that are subsequently investigated in the study (e.g. serogroups, BTFC&YLF genomic groups etc), especially for readers who are not familiar with the field.
Parts of the discussion should already be mentioned in the introduction, e.g. the description of the Finkelstein study. It could then be shortened in the discussion.

**Authors:** We appreciate the reviewer’s thorough feedback and suggestions for improving the manuscript. We have revised the introduction to better cover previous studies on the genetic analysis of *B. pseudomallei*, including both MLST and SNP-based studies. We have also added more information on the environmental prevalence of *B. pseudomallei* and studies comparing patient and environmental isolates. Additionally, we have included descriptions of serogroups and BTFC&YLF genomic groups to provide context for readers unfamiliar with the field. Furthermore, we have moved the description of the Finkelstein study to the introduction to provide better context for our study. This adjustment has allowed us to shorten the discussion section accordingly.

The part of the discussion that refers to the STs of the isolates should be shortened to make it easier to follow.

**Authors:** We have revised the discussion section to make it more concise and easier to follow, focusing on the key findings related to the STs of the isolates.

Line 386: “According to the literature review…” The whole paragraph is missing citations.

**Authors:** Thank you for pointing out the need for citations in this paragraph. We have added the relevant citations to the revised manuscript to support the statements made.

Line 388-391: It is not entirely clear to me whether the authors suggest that relapse, recurrent infection or reinfection can be distinguished by STs? Here, one would probably prefer to use an analyses with higher resolution (?), because as this study shows, certain STs have been present in the environment for a very long time. If I have misunderstood something, please rephrase.

**Authors:** We appreciate the reviewer’s question regarding the use of a higher resolution genotyping technique, such as whole genome SNP analysis, to differentiate between relapse and reinfection. We fully agree with the reviewer’s perspective that this warrants further investigation. Indeed, given that certain STs have been present in the environment for a long time, a comprehensive comparative genomic analysis between clinical and environmental strains sharing the same ST would be invaluable. Such an analysis could provide insights into genetic changes, such as gene acquisition or loss, that have occurred over time. We have rephrased the relevant section in the manuscript to reflect this need for higher resolution analysis and to clarify our stance.

**Reviewer #2:** The manuscript is well-written and organized, with a reliable experimental design. However, a data availability statement needs to be included. It's crucial to provide more details on the in-silico analysis of MLST. The findings are well-supported by solid evidence and conveyed through a well-structured discussion. The study has been conducted rigorously and has produced credible and informative results.
**Authors:** We appreciate the reviewer's positive feedback on the quality and organization of our manuscript. In response to the request for a data availability statement and more details on the *in silico* analysis of MLST, we have made the following revisions to the manuscript:

1. Data Availability Statement:

"The sequencing data of 126 genomes of B. pseudomallei sequenced in this study are available in the GenBank Sequence Read Archive (SRA) under the BioProject accession number PRJNA1113702."

2. Details on In-silico Analysis of MLST:

"Genomic assemblies were performed using programs on Galaxy, a web-based framework for biological computing at the University of Florida. Briefly, genome contigs were generated from the FASTQ files using a Galaxy workflow: FASTQ Groomer (Galaxy Version 1.1.1) and SPAdes genome assembler (Galaxy Version 3.12.0+galaxy1) with default kmers: 21, 35, and 55. Each genome contained 200-300 contigs with approximately 30-50X coverage. The local BLASTN function on BioEdit version 7.0.5.3 was used to search for MLST gene allele sequences [28]. The allele sequences of seven housekeeping genes including, *ace*, *gltB*, *gmhD*, *lepA*, *lipA*, *nark*, and *ndh* of *B. pseudomallei* K96243 (Sequence Type 10), were used as the query sequences. All seven housekeeping gene sequences extracted from these genome data were used in the analysis. New gene allele numbers and new sequence types were assigned by pubMLST (<https://pubmlst.org>). "

These additions and clarifications have been included to enhance the comprehensiveness of the Methods section and to ensure transparency and reproducibility of our study. We hope these revisions meet the reviewer's expectations.

Thank you for your constructive feedback.
--------------------

PLOS authors have the option to publish the peer review history of their article (<a href="<https://journals.plos.org/plosntds/s/editorial-and-peer-review-process#loc-peer-review-history>" target="_blank">what does this mean?</a>). If published, this will include your full peer review and any attached files.

If you choose “no”, your identity will remain anonymous but your review may still be made public.

Do you want your identity to be public for this peer review? For information about this choice, including consent withdrawal, please see our <a href="<https://www.plos.org/privacy-policy>"

Reviewer #1: No

Reviewer #2: No
